# Targeting VEGF with bevacizumab inhibits malignant effusion formation of primary human herpesvirus 8‐unrelated effusion large B‐cell lymphoma in vivo

**DOI:** 10.1111/jcmm.17570

**Published:** 2022-10-09

**Authors:** Fumiya Ogasawara, Tomonori Higuchi, Tomohiro Nishimori, Yumiko Hashida, Kensuke Kojima, Masanori Daibata

**Affiliations:** ^1^ Department of Microbiology and Infection Kochi Medical School, Kochi University Nankoku Japan; ^2^ Department of Hematology Kochi Medical School, Kochi University Nankoku Japan

**Keywords:** bevacizumab, effusion lymphoma, targeted therapy, vascular endothelial growth factor, xenograft model

## Abstract

Primary human herpesvirus 8 (HHV8)‐unrelated effusion large B‐cell lymphoma (ELBCL) is recognized as a new clinical entity, but its pathogenesis and therapeutic strategies remain largely unknown. We have generated two mouse models with profuse lymphomatous effusions that resemble HHV8‐unrelated ELBCL occurring in humans, by grafting the cell lines designated as Pell‐1 and Pell‐2. Using these in vivo models, we evaluated the potential role of vascular endothelial growth factor (VEGF) in the pathogenesis of HHV8‐unrelated ELBCL. Both Pell‐1 and Pell‐2 cells consistently produced very high levels of VEGF in mice, in contrast to in vitro findings of relatively low VEGF production in culture medium by HHV8‐unrelated ELBCL cells, especially Pell‐1 cells. Conversely, returning Pell‐1 cells grown in mice to culture medium markedly suppressed VEGF production to the original in vitro level. These findings suggest that the tumour microenvironment plays a role in the steady production of VEGF. We also found that the interaction between HHV8‐unrelated ELBCL cells and peritoneal mesothelial cells increased the production of VEGF in vitro. Finally, we found that bevacizumab significantly suppressed effusion formation and lymphoma cell growth in both mouse models. These results suggest that bevacizumab is a rational approach to the treatment of HHV8‐unrelated ELBCL.

## 1 INTRODUCTION

Large B‐cell lymphomas that grow exclusively in body cavity effusions without identifiable tumour masses are universally associated with human herpesvirus 8 (HHV8), also called Kaposi sarcoma‐associated herpesvirus, and this lymphoma entity is defined as primary effusion lymphoma (PEL) according to the 2017 World Health Organization classification.^1^ PEL often appears with Epstein–Barr virus (EBV), especially in the setting of human immunodeficiency virus (HIV) infection.^1^ PEL has a characteristic phenotype in that it lacks pan‐B‐cell markers such as CD19 and CD20 but expresses plasma cell markers such as CD138.^1^, ^2^, ^3^

Primary HHV8‐unrelated effusion lymphoma, hereinafter referred to as HHV8‐unrelated effusion large B‐cell lymphoma (ELBCL), has been reported to have features that differ from those of PEL. Most patients with HHV8‐unrelated ELBCL are older and HIV negative, and often have an underlying medical condition that leads to fluid overload.^4^, ^5^, ^6^, ^7^, ^8^ In contrast to PEL, HHV8‐unrelated ELBCL cells express pan‐B‐cell markers and frequently display *c‐MYC* alterations. Studies have concluded that HHV8‐unrelated ELBCL is a clinical entity distinct from PEL.^4^, ^5^, ^6^, ^7^, ^8^ However, because of the limited number of cases reported, there is no standard therapeutic regimen recommended for treatment of the HHV8‐unrelated ELBCL. In these reported cases, most patients received intermediate systemic chemotherapy including CHOP (cyclophosphamide, doxorubicin, vincristine, prednisolone) or a CHOP‐like regimen with or without rituximab, but some had an aggressive clinical course.^5^, ^6^, ^7^ By contrast, a certain proportion of these patients can be managed with drainage alone.^6^, ^7^ These findings suggest the need for agents that target the characteristics of the disease and underpin the importance of controlling the malignant effusions.

Vascular endothelial growth factor (VEGF) is a potent angiogenic factor that was first described as a growth factor for vascular endothelial cells.^9^ VEGF is also a potent inducer of vascular permeability and is implicated in the formation of malignant effusions in cancers such as ovarian cancer and lung cancer.^10^, ^11^, ^12^, ^13^ Most PEL cell lines have been found to produce VEGF constitutively,^14^, ^15^ and HHV8‐encoded viral interleukin‐6 is considered to be a potent inducer of VEGF.^16^ However, knowledge about VEGF production in HHV8‐unrelated ELBCL cells remains limited, and the pathological role of VEGF in the fluid accumulation associated with this lymphoma has not been clarified. To this end, in vivo study models that mimic the tumour microenvironment are desirable because in vitro culture systems do not fully recapitulate all features of the tumour cells that grow in the body cavity effusions. However, the lack of suitable experimental animal models has hindered investigation of the pathogenesis of, and therapeutic strategies for, HHV8‐unrelated ELBCL.

We have previously established an HHV8‐unrelated ELBCL cell line, designated Pell‐1 and generated the first cell line‐derived xenograft (CDX) model that captures the clinical phenomena observed in patients and recapitulates the successive stages of the disease progression.^17^ We have successfully generated a new HHV8‐unrelated ELBCL cell line, Pell‐2 and its CDX model. In this study, using these novel CDX models, we examined the antitumour effects of the anti‐VEGF monoclonal antibody bevacizumab on HHV8‐unrelated ELBCL cells growing in effusions developed in the body cavity of mice, and we describe here the rationale for VEGF‐targeted treatment modalities for this disease.

## 2 MATERIALS AND METHODS

### 2.1 Cell lines and reagents

Four HHV8‐unrelated ELBCL cell lines, STR‐428, OGU1, Pell‐1 and Pell‐2, were used in this study. STR‐428 and OGU1 were obtained from the Japanese Collection of Research Bioresources and Riken BioResource Research Center, respectively.^18^, ^19^ Optimal conditions for the growth of these cell lines were RPMI 1640 medium supplemented with 10% FBS at 37°C in a humidified atmosphere of 5% CO_2_ in air. The detailed characteristics of Pell‐1 are described elsewhere.^17^ In the current study, we established a new cell line, Pell‐2, from the pleural effusion of an HIV‐uninfected Japanese patient with HHV8‐unrelated ELBCL. Derivation of the Pell‐2 cell line was authenticated using short tandem repeat DNA profiling as previously described.^17^ The features of Pell‐2 are summarized in Table S1. Like Pell‐1cells, Pell‐2 cells required 20% FBS for their steady growth, and this cell line was maintained in RPMI 1640 medium containing 20% FBS. The seven PEL cell lines used in this study were obtained from American Type Culture Collection. This study was approved by the Ethics Committee of Kochi Medical School, Kochi University, Japan, and written informed consent was obtained from the patient at the initial diagnosis.

Recombinant human VEGF165 was obtained from R&D Systems. The anti‐VEGF monoclonal antibody bevacizumab and its isotype‐matched mouse IgG_1_ were purchased from Selleck Chemicals and Bio X Cell, respectively.

### 2.2 Quantification of VEGF

The concentrations of human and mouse VEGFs were measured by ELISA using VEGF Quantikine ELISA kits and VEGF DuoSet ELISA kits (both from R&D Systems), respectively. According to the manufacturer's instructions, human and mouse VEGFs do not cross‐react in these assays.

### 2.3 Cell proliferation assay

Cells were seeded into 96‐well plates at a density of 1 × 10^5^/ml (2 × 10^4^ cells/well), and viable cells were counted using a FACSCalibur flow cytometer (Becton Dickinson) by gating out cells stained with propidium iodide.

### 2.4 Quantitative RT–PCR

Real‐time quantitative RT–PCR (qRT–PCR) was used to measure the expression levels of *VEGFR1* and *VEGFR2* mRNAs. Total RNA was extracted using TRIzol reagent (Thermo Fisher Scientific) and Direct‐zol DNA/RNA Miniprep kits (Zymo Research). Total RNA aliquots were treated with DNase to avoid any amplification of genomic DNA and were reverse transcribed using SuperScript VILO cDNA Synthesis kits (Thermo Fisher Scientific). An aliquot of each cDNA was subjected to qRT–PCR analysis as described previously.^20^ The primer sequences are listed in Table S2. Relative gene expression levels were calculated using 2^−ΔCt^ values, with the β‐actin gene (*ACTB*) as a housekeeping control. The PEL cell line BCBL‐1 was included in parallel to determine that expression of *VEGFR1*, but not *VEGFR2*, was detectable by standard RT–PCR.^14^

### 2.5 Flow cytometry

Cells were first stained using 7‐aminoactinomycin D (7‐AAD, BioLegend) to distinguish between live and dead cell populations. Expression of the VEGF receptors VEGFR1 and VEGFR2 was analysed using phycoerythrin (PE)‐labelled anti‐mouse VEGFR1 (clone 49,560, R&D Systems) and PE‐labelled anti‐mouse VEGFR2 (A16085H, BioLegend), respectively. PE‐labelled isotype‐matched antibody (MOPC‐21, BioLegend) was used as a control. Cells were analysed using a BD LSRFortessa flow cytometer (BD Biosciences).

### 2.6 Mouse xenograft model

Nonobese diabetic/SCID (NOD/SCID) mice were purchased from Charles River Laboratories. Cells from cultures were washed twice in serum‐free RPMI 1640 medium, and aliquots of 2 × 10^7^ cells were resuspended in 0.5 ml of the same medium. Six‐week‐old male mice were injected intraperitoneally with a single aliquot of cells. The mice received whole‐body irradiation (250 rad) 1 day before injection with the cells. Euthanized mice were evaluated for the development of effusions and tumours. Viable cells collected from mouse ascites and peritoneal lavage were counted using a flow cytometer by staining with FITC‐labelled anti‐human CD20 antibody (2H7, BioLegend) or FITC‐labelled isotype‐matched control antibody (MPC‐11, BioLegend), after gating out cells stained with 7‐AAD. All experimental protocols were approved by our Institutional Animal Care and Use Committee in compliance with our institutional guidelines and the National Institutes of Health Guide for the Care and Use of Laboratory Animals.

### 2.7 Coculture of peritoneal mesothelial cells

Peritoneal mesothelial cells isolated from human healthy mesentery tissue were provided by Innoprot. The cells were suspended in conditioned medium designated for optimal growth of primary mesothelial cells in vitro (Mesothelial Cell Medium kit, Innoprot) at a density of 2 × 10^5^/ml and grown to confluence in 24‐well plates. After reaching confluence, the medium was removed, the cells were washed with PBS, HHV8‐unrelated ELBCL cells suspended in RPMI 1640 containing 20% FBS were added to the mesothelial cells and the cells were incubated for 3 days.

### 2.8 Statistical analysis

The Mann–Whitney nonparametric *U* test was used to identify differences between pairs of groups. The Kruskal–Wallis ANOVA, followed by Dunn's multiple‐comparison test, were used to compare more than two groups. A *p* value <0.05 was considered to be significant.

## 3 RESULTS

### 3.1 Secretion of VEGF by HHV8‐unrelated ELBCL cell lines in vitro

**FIGURE 1:**
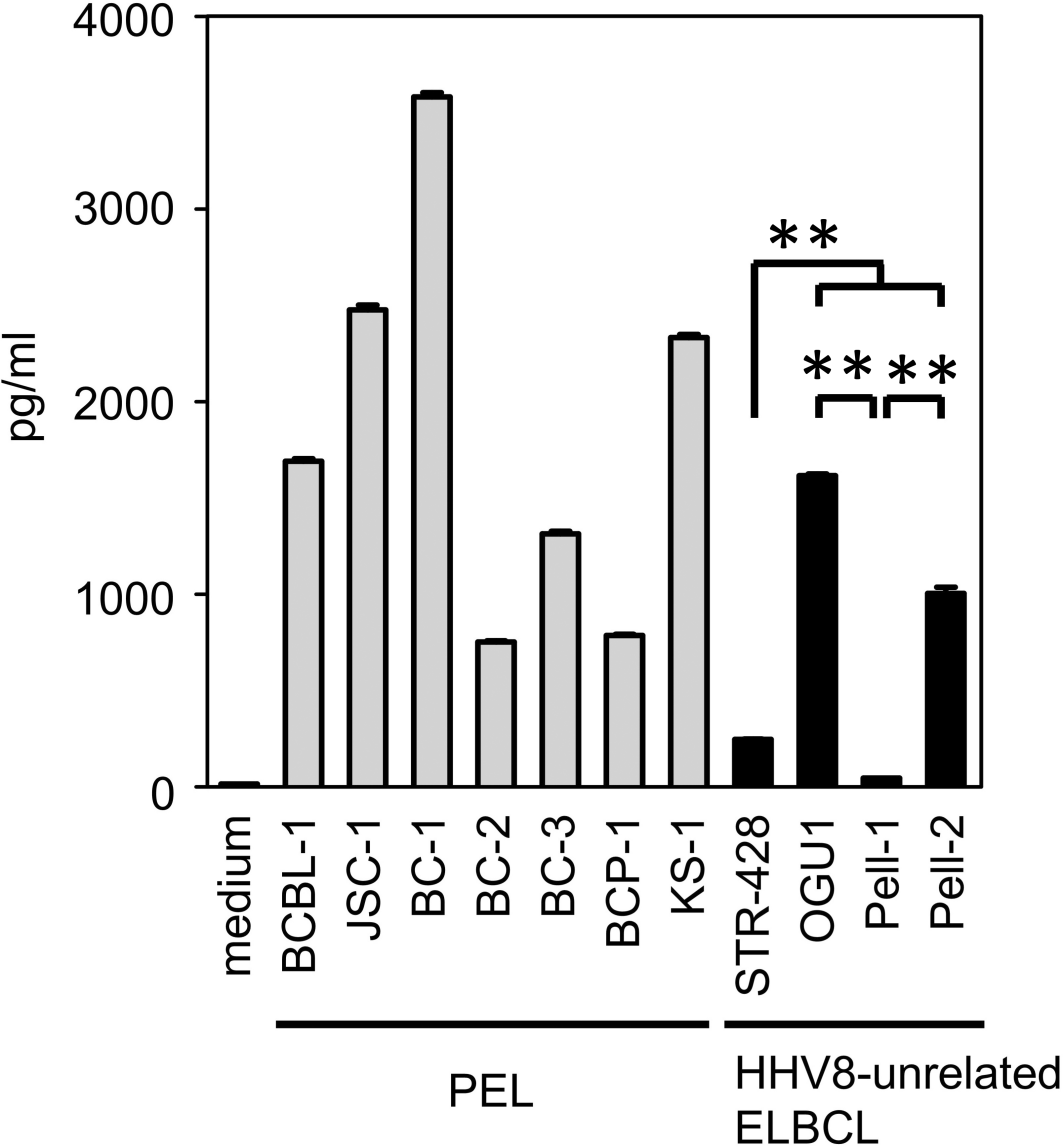
Secretion of vascular endothelial growth factor (VEGF) by primary effusion lymphoma (PEL) and human herpesvirus 8 (HHV8)‐unrelated effusion large B‐cell lymphoma (ELBCL) cell lines in vitro. The cells were seeded in a 24‐well plate at 5 × 10^5^/ml and cultured for 3 days. The concentration of human VEGF in the cell‐free culture supernatants was measured by ELISA. Data are shown as the mean ± SEM (bars) of three independent experiments. Significant differences are shown as **p < 0.01.

We first examined the in vitro secretion of human VEGF by four HHV8‐unrelated ELBCL cell lines and seven PEL cell lines (Figure 1). In line with previous reports,^14^, ^15^ PEL cell lines consistently produced high levels of VEGF. OGU1 and Pell‐2 cells also secreted VEGF at similar levels to those of PEL cell lines: mean concentrations, 1616 and 1006 pg/ml, respectively. By contrast, STR‐428 and Pell‐1 cells secreted only small amounts of VEGF: mean concentrations, 247 and 46 pg/ml, respectively. Thus, the levels of secreted VEGF in vitro differed between HHV8‐unrelated ELBCL cell lines.

### 3.2 Effect of VEGF on HHV8‐unrelated ELBCL cell proliferation in vitro

**FIGURE 2:**
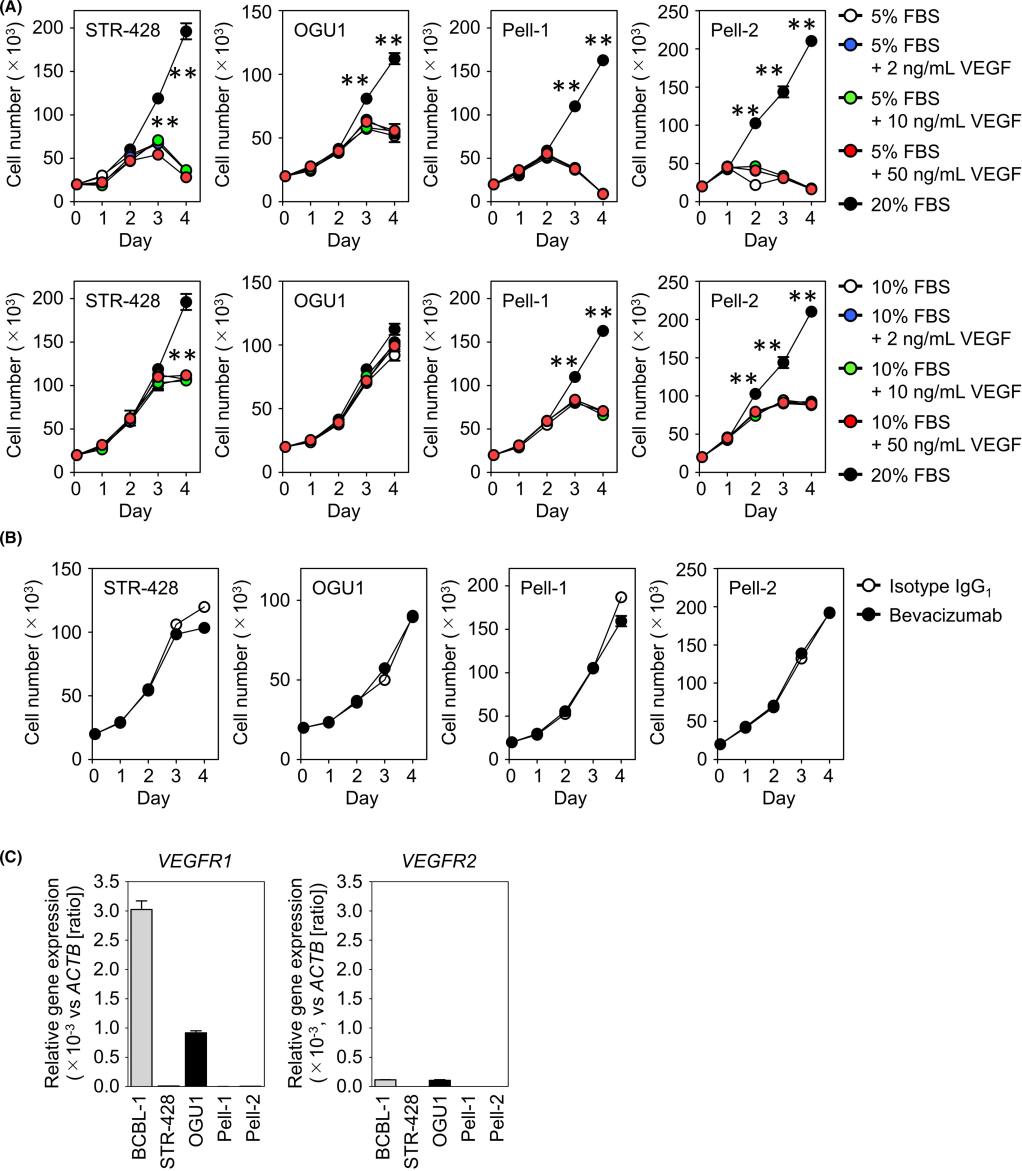
Effect of VEGF on cell growth in vitro and expression of VEGF receptor genes in HHV8‐unrelated effusion ELBCL cell lines. (A) The cells were resuspended at a density of 2 × 10^4^ cells/well in medium supplemented with 20%, 10%, or 5% FBS in the absence or presence of various concentrations of recombinant human VEGF (2–50 ng/ml), and viable cell numbers were counted daily for 4 days. Results from three separate experiments are shown as the mean ± SEM (bars). Significant differences are shown as **p < 0.01. (B) The growing cells in medium with 20% FBS were treated with 100 μg/ml of bevacizumab or isotype control, and cell growth was measured daily for 4 days. Results from three separate experiments are shown. (C) Expression of *VEGFR1* and *VEGFR2* was analysed by qRT–PCR. Relative gene expression levels were calculated using 2^−ΔCt^ values, with the β‐actin gene (*ACTB*) used as a housekeeping control. Results from three separate experiments are shown.

We next evaluated the effect of exogenous VEGF on the growth of HHV8‐unrelated ELBCL cells in vitro. The cells were cultured in the absence or presence of various concentrations of recombinant human VEGF, and cell growth assays were conducted. None of the four HHV8‐unrelated ELBCL cell lines displayed increased proliferation in response to recombinant VEGF when the cells were cultured in medium with lower concentrations of FBS (Figure 2A). We also used bevacizumab, which binds to and neutralizes human VEGF, to examine whether VEGF secreted by HHV8‐unrelated ELBCL cells could contribute to the autocrine growth activity. Bevacizumab did not suppress the cell proliferation in any of the cell lines in vitro (Figure 2B). We next examined the expression of the VEGF receptors, VEGFR1 and VEGFR2. Consistent with the results of a previous report,^19^ the OGU1 cell line expressed *VEGFR1* mRNA and a much lower level of *VEGFR2* mRNA, but these transcripts were not detectable in other cell lines (Figure 2C). VEGFR1 and VEGFR2 proteins were not detected by flow cytometry on the cell surface of these four HHV8‐unrelated ELBCL cell lines (data not shown).

### 3.3 Generation of xenograft models and measurement of VEGF levels in mouse ascites

**FIGURE 3:**
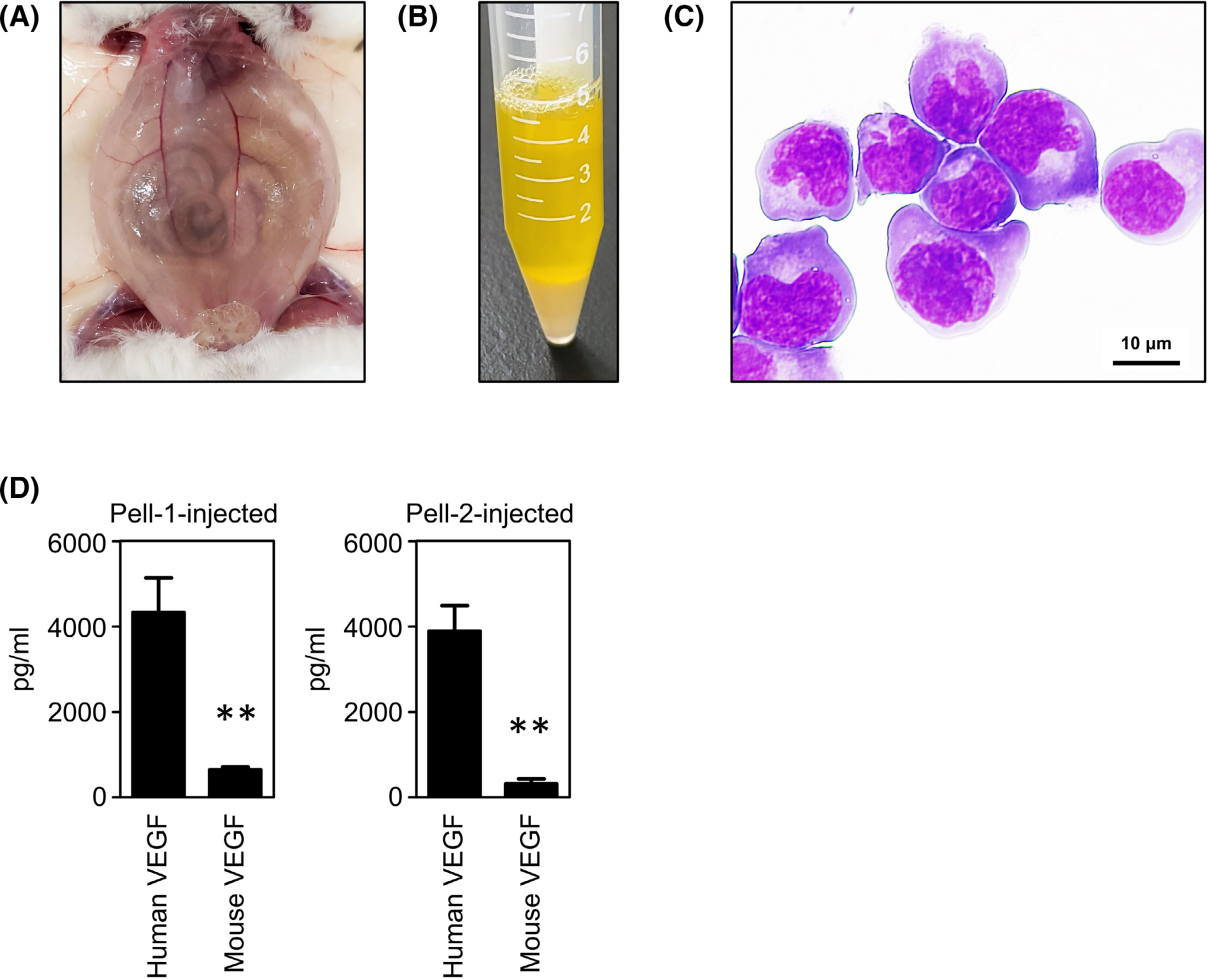
Intraperitoneal xenotransplantation of HHV8‐unrelated effusion ELBCL cells in mice. (A) Photograph of a mouse injected intraperitoneally with Pell‐2 cells (2 × 10^7^) on Day 28 after grafting, showing abdominal distention attributable to the presence of massive ascites. (B) Photograph of ascites collected from the mouse. (C) Cytospin preparation of lymphoma cells in the ascites, showing medium‐to‐large‐sized cells with round or irregular nuclei with slightly coarse chromatin (May–Giemsa staining). (D) Levels of human and mouse VEGFs in ascites from Pell‐1‐ and Pell‐2‐injected mice. Concentrations of VEGFs were measured by ELISA on Day 28 after grafting. Results from three independent experiments are shown as the mean ± SEM (bars). Significant differences are shown as **p < 0.01.

We have previously developed the Pell‐1‐derived xenograft mouse model that closely resembles HHV8‐unrelated ELBCL occurring in humans.^17^ To obtain more CDX models, OGU1, STR‐428 or Pell‐2 cells were injected intraperitoneally into irradiated NOD/SCID mice. Pell‐2 cells gave rise to profuse lymphomatous ascites in all mice tested (*n* = 5) 4 weeks after injection (Figure 3A–C) and subsequent formation of tumours arising from the parietal and/or visceral mesothelial layer of the peritoneum. The mean and median volumes of the ascites were 4.1 and 5.0 ml, respectively. The ascites and peritoneal lavage fluids contained a large number of lymphoma cells, with mean and median total cell numbers of 6.9 × 10^7^ and 6.7 × 10^7^ cells, respectively. The ascitic lymphoma cells had an immunophenotype identical to that of cultured Pell‐2 cells. No lymph node enlargement was found in any of the mice. These findings are consistent with those observed in Pell‐1‐injected mice.^17^ By contrast, neither OGU1‐ nor STR‐428‐injected mice (*n* = 5 for each) developed visible ascites or intraperitoneal tumours even, when observed up to 6 weeks after injection.

Based on these results, we selected Pell‐1 and Pell‐2 cells for further in vivo investigations. We quantified both human and mouse VEGF levels in mouse ascitic fluids on Day 28 after grafting (Figure 3D). The mean and median concentrations of human VEGF were 4329 and 4164 pg/ml for Pell‐1‐injected mice (*n* = 5), respectively, and 3992 and 4475 pg/ml for Pell‐2‐injected mice (*n* = 5), respectively. Mouse VEGF was also detected, but the levels were much lower than those of human VEGF: mean and median concentrations, 650 and 614 pg/ml for Pell‐1‐injected mice, respectively, and 295 and 235 pg/ml for Pell‐2‐injected mice, respectively.

**FIGURE 4:**
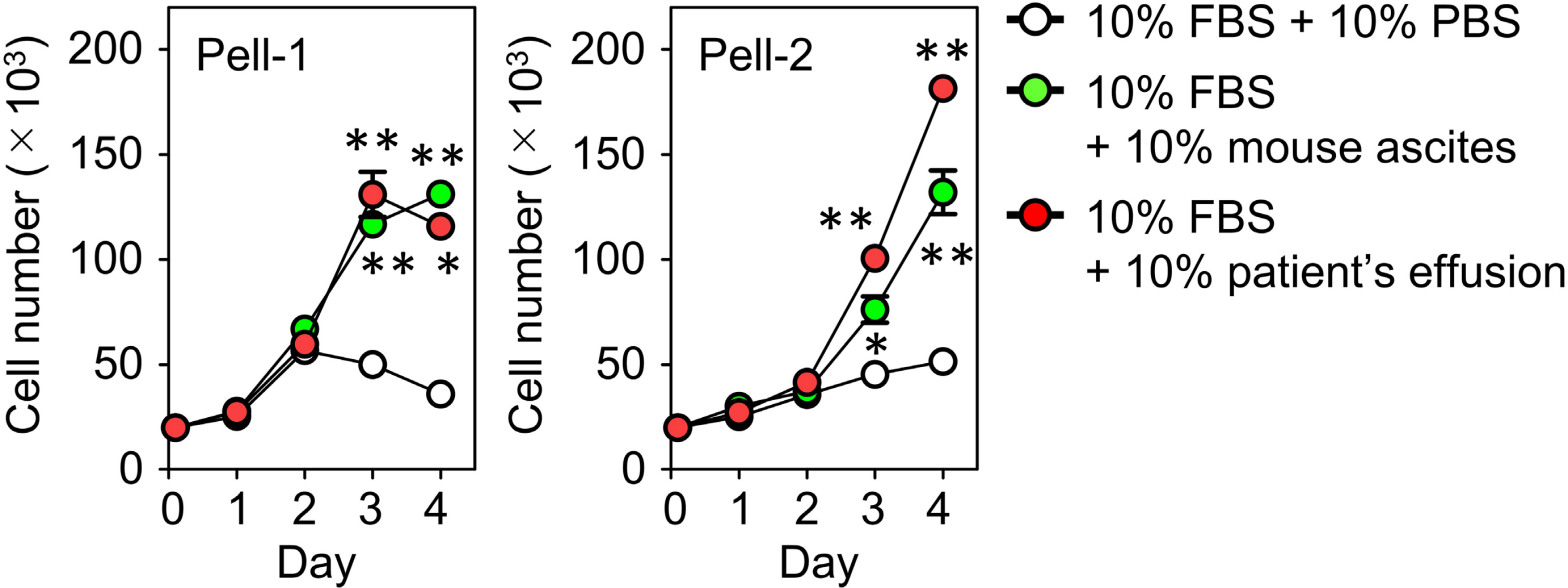
Effect of mouse ascites and body cavity effusions obtained from the patients on proliferation of HHV8‐unrelated effusion ELBCL cells in vitro. The mouse ascites and patients' effusions were centrifuged to remove cellular components. Pell‐1 or Pell‐2 cells were incubated in medium supplemented with 10% FBS in the presence of 10% cell‐free ascitic fluid derived from the cells‐injected mice, 10% cell‐free effusion from each patient, or 10% PBS as a control. Cell growth was measured daily for 4 days. Results from three independent experiments are shown as the mean ± SEM (bars). Significant differences are shown as *p < 0.05, **p < 0.01.

Based on the findings of the accumulation of large amounts of human VEGF in ascites from both Pell‐1‐ and Pell‐2‐injected mice, we next assessed the effect of the mouse ascites on cell proliferation in vitro. Pell‐1 or Pell‐2 cells were cultured in medium with 10% FBS in the presence of 10% cell‐free mouse ascitic fluid obtained from the respective cell‐injected mice or 10% PBS as a control. Compared with the controls, addition of the ascites significantly promoted both Pell‐1 and Pell‐2 cell growth (Figure 4). We also assessed the effect of the cell‐free effusion obtained from each patient on cell proliferation in vitro. The patient's effusion significantly increased both Pell‐1 and Pell‐2 cell growth (Figure 4). We found a high concentration of VEGF (mean 3786 pg/ml) in the patient's effusion from which Pell‐1 was established but failed to evaluate the level of VEGF in the sample from which Pell‐2 was established due to insufficient volume. Because these cell lines do not express VEGFRs, these results raise the possibility that certain growth factors contained in mouse ascites and patient's effusion, other than VEGF/VEGFR signals, supported the cell growth.

### 3.4 Reevaluation of VEGF secretion level and VEGFR expression using HHV8‐unrelated ELBCL cells recovered from mice

**FIGURE 5:**
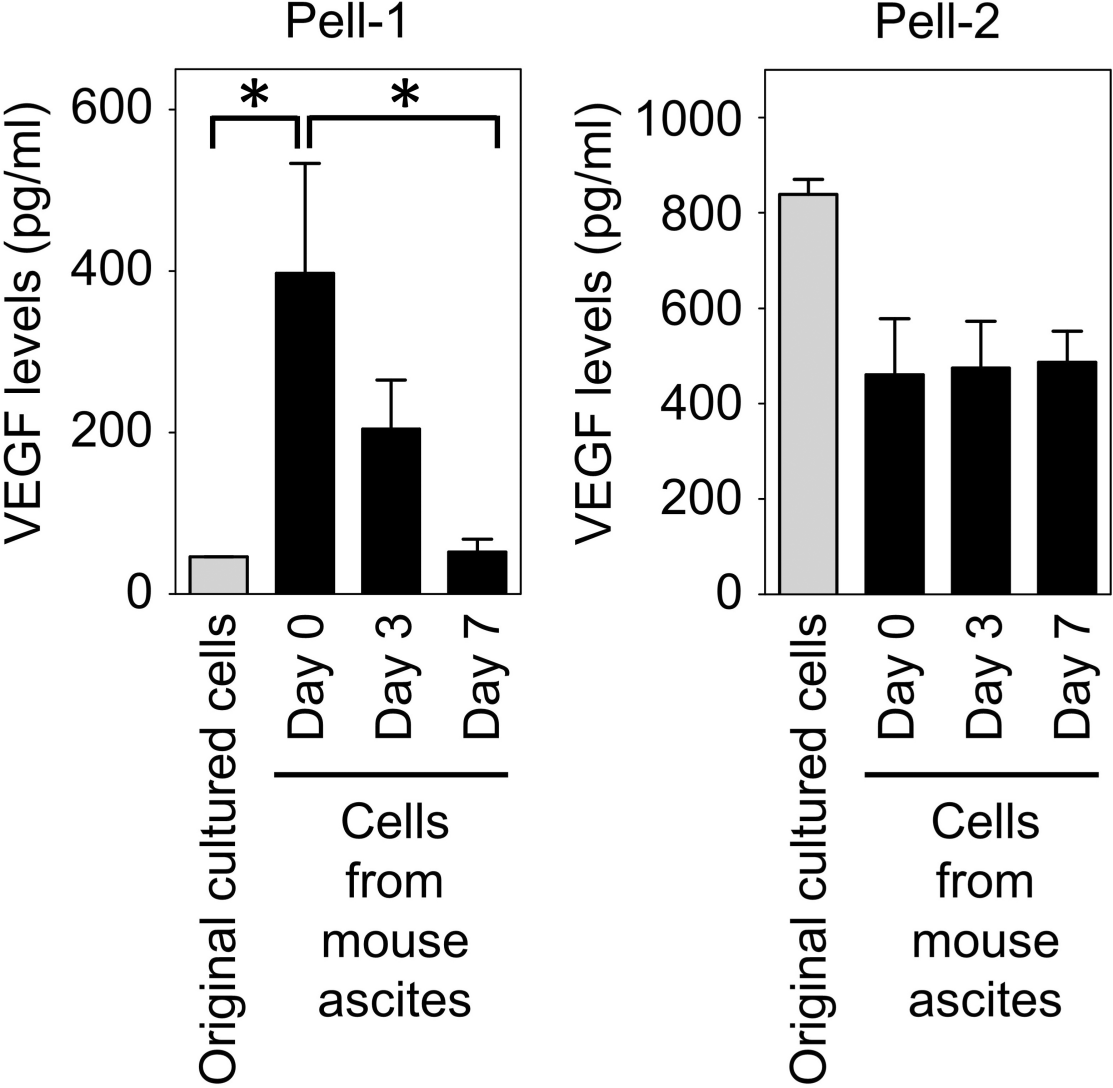
Time course of VEGF secretion after recultivation of lymphoma cells recovered from mouse ascites. Pell‐1 and Pell‐2 cells were collected from the ascites of their respective mice, washed with RPMI 1640 medium, and cultured in medium with 20% FBS for 0, 3 or 7 days. The cells were harvested on each day, resuspended in fresh medium at a density of 5 × 10^5^/ml and incubated for a further 3 days. Human VEGF concentration in the culture supernatants was measured by ELISA. The original cultured Pell‐1 and Pell‐2 cells that had been used for injection were also incubated for 3 days followed by VEGF quantification. Results from three independent experiments are shown as the mean ± SEM (bars). Significant differences are shown as *p < 0.05.

We next investigated whether the capacity for VEGF production acquired in mice was transient or retained when the recovered lymphoma cells were recultured in medium. Pell‐1 and Pell‐2 cells collected from ascitic fluids were incubated in conditioned medium for 0, 3 or 7 days. Each type of harvested cell was resuspended in fresh medium and incubated for a further 3 days, and human VEGF production was quantified. Large amounts of VEGF were detected in the supernatants of Pell‐1 cells when measured immediately after recovery from ascites, but the levels decreased with incubation time and reached the original in vitro baseline levels after 7 days of culture (Figure 5). By contrast, the recovered Pell‐2 cells secreted VEGF constitutively at levels similar to that in the original cultured Pell‐2 cells even after the 7‐day culture. These results suggest that the in vivo circumstance is essential for substantial VEGF production by HHV8‐unrelated ELBCL cells, especially Pell‐1 cells, which do not produce high levels of VEGF in vitro. Consistent with the in vitro studies, Pell‐1 and Pell‐2 cells freshly collected from the mice expressed neither VEGFR1 nor VEGFR2 at both the mRNA and protein levels (data not shown).

### 3.5 Effect of coculture of mesothelial cells and HHV8‐unrelated ELBCL cells on VEGF production

**FIGURE 6:**
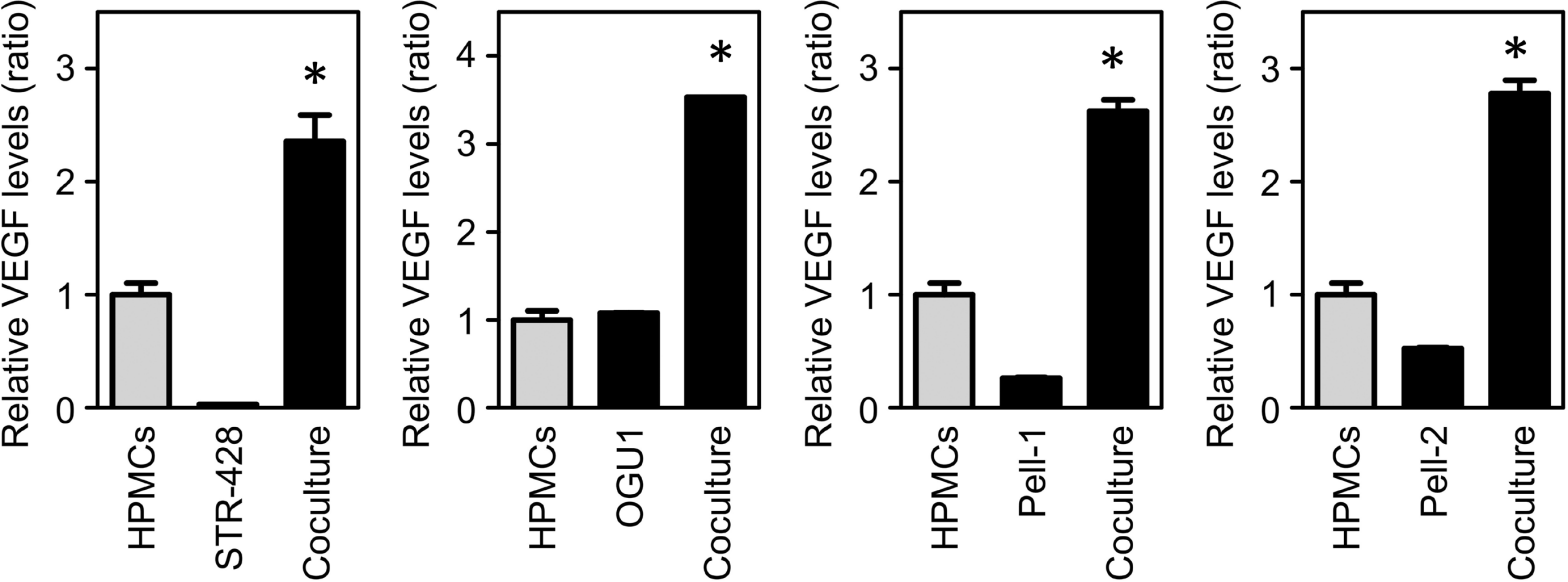
Effect of coculture of mesothelial cells and HHV8‐unrelated ELBCL cells on VEGF production in vitro. STR‐428, OGU1, Pell‐1 or Pell‐2 cells were cocultured with confluent human peritoneal mesothelial cells in RPMI 1640 medium supplemented with 20% FBS for 3 days. The graphs show VEGF levels in cultures of the cell lines and in cocultures of the cell lines and mesothelial cells relative to those in cultures of mesothelial cells alone. Results from three independent experiments are shown as the mean ± SEM (bars). Significant differences are shown as *p < 0.05. HPMCs, human peritoneal mesothelial cells.

Mesothelial cells can produce VEGF, which is thought to contribute to the accumulation of malignant ascites.^21^, ^22^, ^23^ We next investigated whether VEGF production by HHV8‐unrelated ELBCL cells would be augmented by the presence of human peritoneal mesothelial cells. After a 3‐day incubation of confluent mesothelial cells, the mean concentration of VEGF was 1500 pg/ml. Coculture of HHV8‐unrelated ELBCL cells with the mesothelial cells resulted in a synergistic increase in VEGF production (Figure 6). These findings imply that mesothelial cells play a role in modulating VEGF production in the tumour microenvironment. However, the mesothelial cells did not influence cell growth of Pell‐1 and Pell‐2 cells in this culture condition (data not shown).

### 3.6 Effect of bevacizumab on suppression of HHV8‐unrelated ELBCL in vivo

**FIGURE 7:**
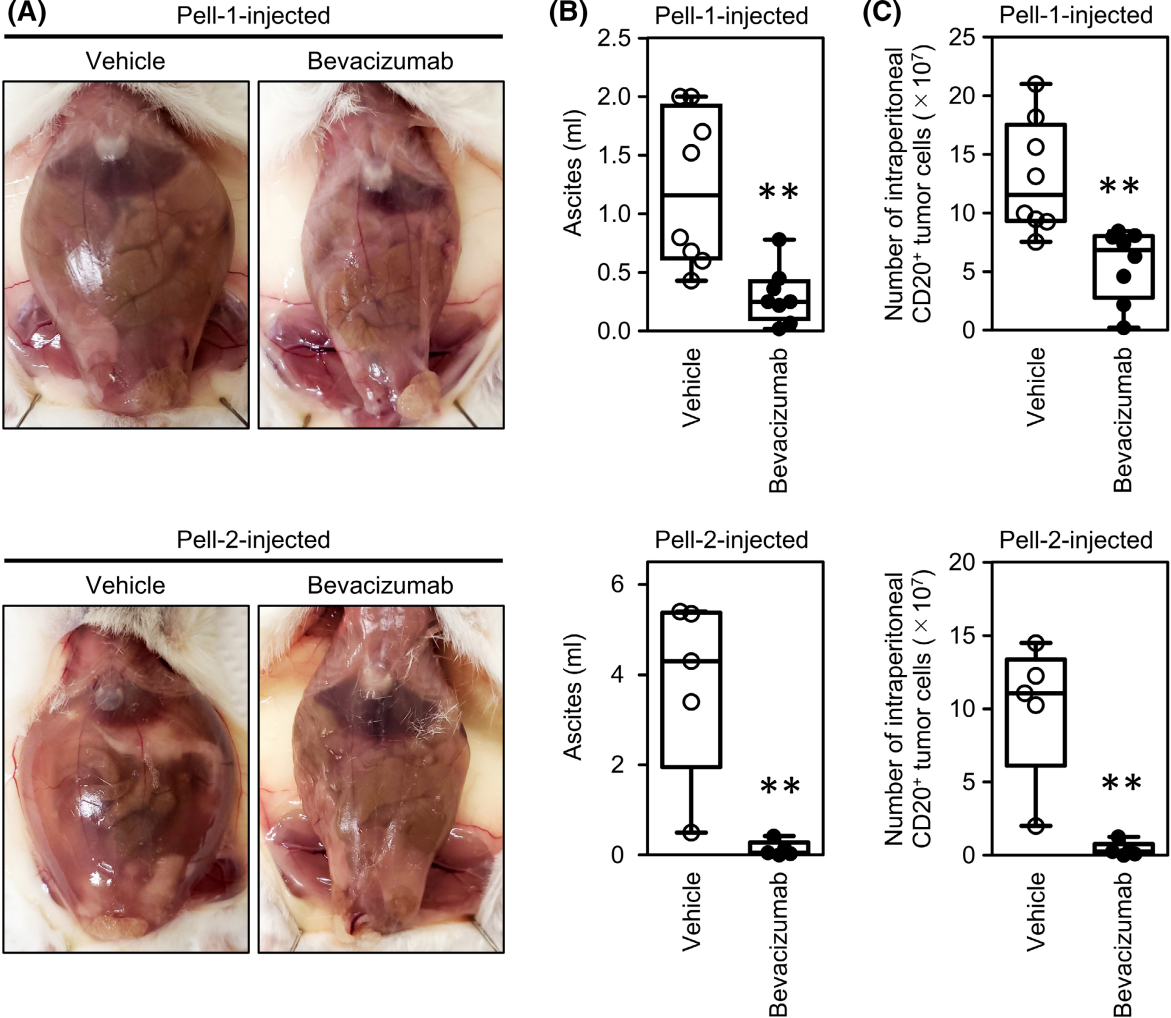
Effect of bevacizumab treatment on suppression of HHV8‐unrelated ELBCL in vivo. Pell‐1 or Pell‐2 cells (2 × 10^7^/mouse) were injected into the peritoneal cavity of irradiated NOD/SCID mice. On Day 4 after injection, the mice were divided into two groups and treated with bevacizumab (100 μg/mouse) or vehicle (PBS) once daily, 3 days per week. Mice were euthanized on Day 28 after grafting. (A) Representative photographs of a mouse treated with vehicle alone versus one treated with bevacizumab. (B) Box plots show the ascites volumes in mice treated with bevacizumab versus those treated with vehicle alone. (C) Box plots show the number of lymphoma cells in the two groups. Cells recovered from ascites and peritoneal lavage fluids were stained with an anti‐human CD20 antibody, and CD20‐positive cells were counted using flow cytometry. Horizontal lines within the boxes indicate median values, and bars indicate maximum and minimum values of the results from eight Pell‐1‐injected mice or from five Pell‐2‐injected mice per group. Significant differences are shown as **p < 0.01.

Finally, we used bevacizumab to investigate the effect of VEGF on HHV8‐unrelated ELBCL progression in vivo. Mice injected with Pell‐1 or Pell‐2 cells were randomly divided into two groups on Day 4 after injection and treated intraperitoneally with bevacizumab or vehicle. The experiments were performed three times independently with a total of eight mice/group for Pell‐1‐injected mice and five mice/group for Pell‐2‐injected mice. We measured ascites volume and the numbers of lymphoma cells in ascites and peritoneal lavage fluids in all mice at autopsy on Day 28 after grafting. The bevacizumab‐treated mice showed no signs or symptoms of toxicity that indicate stress and pain, such as progressive weight loss or behavioural changes, throughout the course of the therapy. In both Pell‐1‐ and Pell‐2‐injected mice, bevacizumab treatment significantly reduced ascites volume compared with vehicle injection (*p* = 0.004 and *p* = 0.008, respectively; Figure 7A,B). Significantly fewer lymphoma cells were found in the bevacizumab‐treated mice (*p* = 0.001 and *p* = 0.008, respectively; Figure 7C). Notably, bevacizumab inhibited ascites formation almost completely, as evidenced by ascites volumes <0.1 ml in four of five (80%) Pell‐2‐injected mice, whereas this therapeutic effect was seen only in two of eight (25%) Pell‐1‐injected mice. Although the therapeutic efficacy appears to differ between cell types, these suggest that bevacizumab significantly suppressed the progression of HHV8‐unrelated ELBCL by preventing fluid accumulation in vivo.

## 4 DISCUSSION

During the past decade, primary effusion lymphomas lacking HHV8 have been documented, and terms such as PEL‐like lymphoma, HHV8‐unrelated PEL, and HHV8‐negative effusion‐based lymphoma (EBL) have been coined to describe these conditions. However, the terms ‘PEL’ and ‘EBL’ seem to have been used interchangeably in the literature.^8^ The recent consensus opinion of an international group of hematopathologists is that the designation PEL should be restricted to cavity‐based lymphomas with neoplastic HHV8‐infected cells.^24^ Accordingly, we designated the HHV8‐negative cases as primary HHV8‐unrelated ELBCL in this study. Intriguingly, in these documented cases, patients of East Asian origin account for about 60% of cases of HHV8‐unrelated ELBCLs.^5^, ^6^, ^7^, ^8^, ^25^, ^26^, ^27^, ^28^, ^29^, ^30^, ^31^

Cell lines provide invaluable tools for research on rare diseases such as HHV8‐unrelated ELBCL. To the best of our knowledge, to date, only three HHV8‐unrelated ELBCL cell lines, STR‐428, OGU1 and Pell‐1, all of which were derived from Japanese patients, have been characterized in detail.^17^, ^18^, ^19^ Using these HHV8‐unrelated ELBCL cell lines together with a newly established Pell‐2 cell line, we assessed for the first time the role of VEGF in lymphomagenesis both in vitro and in vivo, and efficacy of a VEGF‐targeted strategy for this neoplasm. We found two different VEGF secretion patterns by HHV8‐unrelated ELBCL cells in vitro. OGU1 and Pell‐2 cells produced large amounts of VEGF, but STR‐428 and Pell‐1 cells released only small amounts of this protein into their culture medium. None of these HHV8‐unrelated ELBCL cell lines, except OGU1 in which only *VEGFR1* transcript was detected, expressed VEGFRs. Exogenous VEGF did not stimulate their proliferation, nor did bevacizumab inhibit their cell growth in culture. These studies suggest that VEGF is not an autocrine growth factor for HHV8‐unrelated ELBCL cells in vitro.

Cell culture systems provide limited information because of the different microenvironment from that in vivo. In our study, Pell‐1 and Pell‐2 cells produced effusion lymphomas with prominent ascites when injected into the peritoneal cavity of irradiated NOD/SCID mice. A major strength of this study is that we successfully generated two xenograft models, both of which resemble human HHV8‐unrelated ELBCL and provide the in vivo microenvironment for examining the underlying mechanisms more precisely. In vivo, very high levels of human VEGF were found in ascitic fluids from mice injected with Pell‐1 and Pell‐2 cells. Of note, although Pell‐1 cells produced low amounts of VEGF in vitro, a marked increase in its level was observed in the ascites of Pell‐1‐injected mice. Conversely, when Pell‐1 cells growing in mouse ascites were returned to culture medium, VEGF secretion was suppressed to the original level. These results suggest that VEGF production by HHV8‐unrelated ELBCL cells is activated in vivo by the tumour environment despite its suppression in vitro. Taken together, our data suggest that stimulation of vascular permeability by VEGF is responsible for the fluid accumulation in HHV8‐unrelated ELBCL in vivo.

Pell‐1 and Pell‐2 cells showed continuous growth in mouse ascites in our study. VEGF can directly induce activation of VEGFR2, a principal VEGF signalling receptor, which leads to the activation of downstream signalling molecules in a cell proliferation pathway.^32^, ^33^ Because the dependence on the signalling pathway in tumour cells may be altered by the microenvironment,^34^ we examined whether Pell‐1 and Pell‐2 cells acquire the expression of VEGFRs during growth in mice. However, consistent with in vitro findings, VEGFR1 and VEGFR2 were not expressed in vivo. This finding suggests that VEGF alone does not act as a critical autocrine growth factor that regulates proliferation of HHV8‐unrelated ELBCL cells in vivo.

Intriguingly, we found that incubation with the mouse ascitic fluid and body cavity effusions obtained from the patients significantly promoted cell proliferation of Pell‐1 and Pell‐2 cells in vitro. It is plausible that certain autocrine growth factors produced by HHV8‐unrelated ELBCL cells in vivo and/or paracrine growth factors produced by stroma cells following interaction with tumour cells may contribute to the promotion of tumour proliferation. Malignant ascites serves as a reservoir for a complex mixture of soluble factors and cellular components, which provide a local tumour‐promoting microenvironment for tumour cells, especially in ovarian cancer.^35^, ^36^ It is well known that stroma cells play a significant role in malignant progression in many tumours. Mesothelial cells, which line the body's serous cavities, are considered to be one of the central elements controlling tumour progression because they can secrete various growth factors constitutively or in response to inflammatory stimuli. We therefore hypothesized that mesothelial cells generate an environment that confers a proliferative advantage to HHV8‐unrelated ELBCL cells and contributes to the increased accumulation of VEGF in vivo. The current study provides evidence that the interaction between HHV8‐unrelated ELBCL cells and peritoneal mesothelial cells increased the production of VEGF and highlights the importance of the tumour microenvironment for the efficient production of VEGF. However, supportive activity for HHV8‐unrelated ELBCL cell proliferation by mesothelial cells was not evident in our in vitro cell growth assay.

A further strength of this study is that we found a prominent role of VEGF in sustaining HHV8‐unrelated ELBCL growth in the blocking experiments using bevacizumab in the mouse models. In these models, accumulation of ascites was significantly prevented in bevacizumab‐treated mice compared with vehicle‐treated mice. Previous studies have shown that in mice bearing ovarian tumours, bevacizumab/A4.6.1 reduced the accumulation of malignant ascites but had only a limited effect on the tumour burden.^13^, ^37^ Our finding that bevacizumab also significantly inhibited intraperitoneal growth of HHV8‐unrelated ELBCL may be important to the therapeutic strategy. Because VEGF does not exert autocrine growth factor activity in HHV8‐unrelated ELBCL, bevacizumab appears not to act as a direct cytostatic agent.

The current study suggests that bevacizumab suppresses fluid retention essentially by blocking vascular permeability caused by VEGF, which consequently inhibits the growth of HHV8‐unrelated ELBCL because body cavity effusions provide a sanctuary for this type of lymphoma that affords a survival advantage. Thus, controlling lymphomatous effusion seems to be a reasonable strategy in the treatment of HHV8‐unrelated ELBCL. A certain proportion of patients can be managed with drainage alone.^6^, ^7^ However, it seems unlikely that bevacizumab monotherapy is fully effective for eradicating the disease in all cases. In our xenograft models, bevacizumab almost completely inhibited ascites production in Pell‐2‐injected mice but did so to a lesser extent in mice injected with Pell‐1 cells that initially secreted small amounts of VEGF. These findings raise two questions that should be explored by further studies. The first is whether the VEGF content in effusions can be used to predict the antitumour effect of bevacizumab in HHV8‐unrelated ELBCL. The second is whether the antitumour effect of bevacizumab can be improved by combining with cytotoxic agents or with agents targeting other molecules such as c‐MYC.^17^

Our study has some limitations. First, we cannot explain why mice injected with STR‐428 or OGU1 cells developed neither noticeable ascites nor peritoneal tumours, even though these can grow in culture medium with lower concentrations of FBS than that used in the culture of Pell‐1 and Pell‐2 cells. Second, we did not identify the endogenous signals that regulate HHV8‐unrelated ELBCL growth in connection with VEGF signal transduction. Finally, we do not know whether bevacizumab can also induce regression of malignant effusions established in mice. The antitumour effect of intravenous administration of bevacizumab also remains to be elucidated.

In summary, using CDX mouse models that are equivalent to human HHV8‐unrelated ELBCL, we provide evidence that these lymphoma cells secrete high levels of VEGF in vivo. The current study also shows that VEGF plays a critical role in the pathogenesis of HHV8‐unrelated ELBCL because bevacizumab suppressed lymphoma cell growth by preventing effusion formation in mice. Most patients with HHV8‐unrelated ELBCL are elderly or have comorbidities that cause fluid overload in body cavities, which may hamper the use of aggressive chemotherapy. The results presented here suggest that bevacizumab, one of the most widely used therapeutics in oncology, represents a rational approach to the treatment of HHV8‐unrelated ELBCL.

## AUTHOR CONTRIBUTIONS

**Fumiya Ogasawara** involved in investigation (equal); resources (equal) and writing—original draft (supporting). **Tomonori Higuchi** contributed to conceptualization (equal); formal analysis (lead); funding acquisition (equal); investigation (equal); methodology (equal) and visualization (lead). **Tomohiro Nishimori** and **Yumiko Hashida** performed investigation (equal). **Kensuke Kojima** collected resources (equal). **Masanori Daibata** performed conceptualization (equal); funding acquisition (equal); methodology (equal); project administration (lead); supervision (lead); writing—original draft (lead) and writing—review and editing (lead).

## CONFLICT OF INTEREST

The authors confirm that there are no conflicts of interest.

## Supporting information


Table S1

Table S2

## Data Availability

The data that support the findings of this study are available from the corresponding author upon reasonable request.
