# Supplementary material for: Targeting VEGF with bevacizumab inhibits malignant effusion formation of primary human herpesvirus 8‐unrelated effusion large B‐cell lymphoma in vivo
Source: J Cell Mol Med. 2022 Oct 9;26(22):5580–9. doi: 10.1111/jcmm.17570 (PMC9667516; doi:10.1111/jcmm.17570)
Supplement: Supplementary file 1 — Table S1 [file JCMM-26-5580-s002.docx]

**TABLE S1** Synopsis of data on the Pell-2 cell line

| **Parameter** | **Features** |
| --- | --- |
| Clinical data |  |
| Patient | 76-year-old Japanese man |
| Diagnosis | Primary human herpesvirus 8-unrelated effusion large B-cell lymphoma |
| Specimen | Pleural effusion |
| Specimen collection  Year of establishment | At diagnosis  2021 |
| Cell line |  |
| Culture medium | RPMI 1640 + 20% fetal bovine serum |
| Growth pattern | Single cells in suspension |
| Doubling time | 25 hours |
| Optimal cell density | 7 × 10^5^ cells/ml |
| Optimal split | 1:4 every 3–4 days |
| Cryopreservation | In CELLBANKER^TM^ (Takara Bio, Japan) |
| Morphology | Medium-to-large-sized cells with round or irregular nuclei |
| Viral status | Negative for human herpesvirus 8 and Epstein–Barr virus |
| Immunoprofile | Positive for CD19, CD20, CD22, and CD79a  Negative for CD138 and T-cell markers |
| Karyotypic analysis  Gene rearrangement | 47, X, -X or -Y, add(1)(q21), add(13)(q22), +der(?)t(?;1)(?;q21), +mar  Positive for *c-MYC* rearrangement  Negative for *BCL2* and *BCL6* rearrangements |
| Tumorigenic capacity | Intraperitoneal growth in irradiated (250 rad) NOD/SCID mice |
| Authentication | Yes, by short tandem repeat DNA fingerprinting |
