# Supplementary material for: Targeting VEGF with bevacizumab inhibits malignant effusion formation of primary human herpesvirus 8‐unrelated effusion large B‐cell lymphoma in vivo
Source: J Cell Mol Med. 2022 Oct 9;26(22):5580–9. doi: 10.1111/jcmm.17570 (PMC9667516; doi:10.1111/jcmm.17570)
Supplement: Supplementary file 2 — Table S2 [file JCMM-26-5580-s001.docx]

**TABLE S2** Sequences of primers used for real-time qRT–PCR

| **Target**  **region** | **Primer name** | **Sequence (5′→3′)** | **Nucleotide position** | **Accession number** |
| --- | --- | --- | --- | --- |
| *VEGFR1* | VEGFR1-F | ACCGAATGCCACCTCCATG | 3981–4087 | NM_002019.4 |
|  | VEGFR1-R | AGGCCTTGGGTTTGCTGTC |  |  |
| *VEGFR2* | VEGFR2-F | ATGGTTCTTGCCTCAGAAGAGCTG | 4086–4206 | NM_002253.4 |
|  | VEGFR2-R | TCTGGTTTGAGCCTTCAGATGCC |  |  |
| *β-actin* | ACTB-F | GTGGATCAGCAAGCAGGAGTATGA | 1257–1342 | NM_001101.4 |
|  | ACTB-R | TAGGTTTTGTCAAGAAAGGGTGTAA |  |  |
